# Stronger neural response to canonical finger‐number configurations in deaf compared to hearing adults revealed by FPVS‐EEG

**DOI:** 10.1002/hbm.26297

**Published:** 2023-04-06

**Authors:** Margot Buyle, Aliette Lochy, Valentina Vencato, Virginie Crollen

**Affiliations:** ^1^ Psychological Sciences Research Institute (IPSY) and Institute of NeuroScience (IoNS), Université Catholique de Louvain Louvain‐la‐Neuve Belgium; ^2^ Department of Behavioural and Cognitive Sciences, Faculty of Humanities, Social and Educational Sciences Institute of Cognitive Science and Assessment, University of Luxembourg Esch‐sur‐Alzette Luxembourg; ^3^ Present address: Center for Neural Science, New York University New York New York 10003 USA

**Keywords:** canonical representations, deafness, electroencephalography (EEG), finger‐number configurations, frequency‐tagging, sign language

## Abstract

The linguistic counting system of deaf signers consists of a manual counting format that uses specific structures for number words. Interestingly, the number signs from 1 to 4 in the Belgian sign languages correspond to the finger‐montring habits of hearing individuals. These hand configurations could therefore be considered as *signs* (i.e., part of a language system) for deaf, while they would simply be number *gestures* (not linguistic) for hearing controls. A Fast Periodic Visual Stimulation design was used with electroencephalography recordings to examine whether these finger‐number configurations are differently processed by the brain when they are *signs* (in deaf signers) as compared to when they are *gestures* (in hearing controls). Results showed that deaf signers show stronger discrimination responses to canonical finger‐montring configurations compared to hearing controls. A second control experiment furthermore demonstrated that this finding was not merely due to the experience deaf signers have with the processing of hand configurations, as brain responses did not differ between groups for finger‐counting configurations. Number configurations are therefore processed differently by deaf signers, but only when these configurations are part of their language system.

## Graphical abstract

Deaf signers show stronger discrimination responses to canonical finger‐montring configurations compared to hearing controls. Sign language knowledge seems to shape the neural correlates underlying finger‐number configurations.

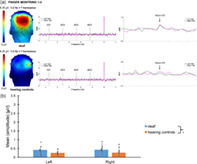

## 1 INTRODUCTION

Humans possess a grasp of quantitative concepts that has presumably developed independently of, and before, language (Butterworth, 1999; Carey, 1998; Dehaene, 1997; Dehaene, 2001; Wynn, 1998). This ability is considered as the root of the approximate representation of *non‐symbolic* quantity and has been attributed to the so‐called approximate number system (ANS) (Barth et al., 2008; Barth et al., 2006; Barth et al., 2005; Dehaene, 2011; Feigenson et al., 2004). Besides this *non‐symbolic* representation of numbers, a variety of specific numerical codes such as number words (one, two, three, etc.) and Arabic numerals (1, 2, 3, etc.) has also to be mastered. This *abstract* learning is strongly anchored in our language system (Miller et al., 1995), and requires children to progressively move from concrete mathematical skills based on physical objects towards a more symbolic mathematical ability focused on numerals (Kolkman et al., 2013).

Over the last decades, it has been assumed that canonical finger configurations could facilitate this process by providing concrete referents to abstract number symbols (Butterworth, 1999; Gunderson et al., 2015; Soylu et al., 2018; Van den Berg et al., 2022). These canonical finger configurations include finger‐counting and finger‐montring gestures, which are typically used in a given culture to respectively count and show numerosities with the fingers. Finger‐counting and finger‐montring therefore differ in terms of purposes (i.e., counting for finger‐counting versus communicating a quantity for finger‐montring), but also differ in terms of handshape specificities. In many European countries, the thumb and the index fingers are, for example, successively raised to count to two (finger‐counting), while the index and middle fingers are simultaneously raised to communicate about the same quantity (finger‐montring). Despite these specificities, finger‐counting and finger‐montring nevertheless both allow a direct recognition of the magnitude they represent (Di Luca & Pesenti, 2008). Support for this latter assumption can be found in: (1) behavioural studies showing that canonical finger configurations are more efficiently enumerated and processed than other (non‐canonical) finger configurations (Di Luca et al., 2006; Di Luca & Pesenti, 2008; Lafay et al., 2013; Marlair et al., 2021; Noël, 2005; Soylu et al., 2019; van den Berg et al., 2021; Van den Berg et al., 2022), and (2) brain research demonstrating that canonical finger configurations induce higher activation of occipital and parietal brain regions (i.e., the identified brain substrate of number‐selective representations: (S. Dehaene & Cohen, 2007; S. Dehaene et al., 2003; Nieder & Dehaene, 2009)) than non‐canonical representations (Marlair et al., 2021; Proverbio & Carminati, 2019; Soylu et al., 2019; van den Berg et al., 2021, Van den Berg et al., 2022).

**FIGURE 1:**
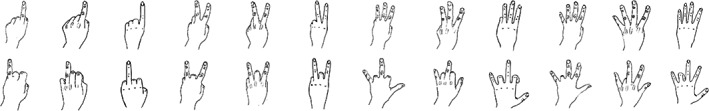
Stimuli that were used in this experiment (finger‐montring 1 to 4). First line represents the canonical configurations. Second line represents the non‐canonical ones. Three different drawing designs (and their mirror images) were used for each numerosity.

Due to their frequent use in early number learning and daily communication, culture‐specific number gestures therefore become semantically associated to other (non‐symbolic and abstract) number representations (van den Berg et al., 2021). These associations are weaker with non‐canonical number gestures (due to their non‐familiarity), but may in contrast increase when finger configurations are embedded in a language system such as sign language. In Belgium, the number signs from 1 to 5 interestingly correspond to the finger‐montring habits of hearing individuals (see Figure 1). As research has shown that deaf signers develop specific cognitive processes for perceiving fingers and hand configurations (Baker et al., 2005; Emmorey et al., 2003; Muir & Richardson, 2005), we could therefore hypothesise that deaf signers will process these configurations differently than hearing controls.

To investigate this question, brain responses to number gestures were compared in deaf signers and hearing controls with a highly sensitive and implicit method combining frequency‐tagging and electroencephalography (EEG) (i.e., Fast Periodic Visual Stimulation, FPVS) (Retter & Rossion, 2016; Rossion, 2014). FPVS consists in presenting a category of stimuli at a frequency X, which triggers a general visual response in the brain, measurable at exactly that frequency X. Within this periodic presentation, another category of stimuli (deviant) is inserted at a frequency Y. If the deviants are discriminated, then a measurable neural response occurs at the frequency of stimulation Y for this category of stimuli. This method provides an objective marker of category discrimination and has already been successfully applied to the discrimination of visual quantities (Guillaume et al., 2018; Marlair et al., 2021), digits grouped on the basis of parity (odd/even) or magnitude (small/big) (Guillaume et al., 2020; Marinova et al., 2021), or digits among letters (Lochy & Schiltz, 2019). It has also been successfully used to investigate visual processing and brain reorganisation in deaf individuals (Benetti et al., 2017; Bottari et al., 2020; Gwinn & Jiang, 2020; Retter et al., 2019; Stroh et al., 2022) and is therefore particularly adapted to unravel changes associated with number categorizations in deaf signers.

**FIGURE 2:**
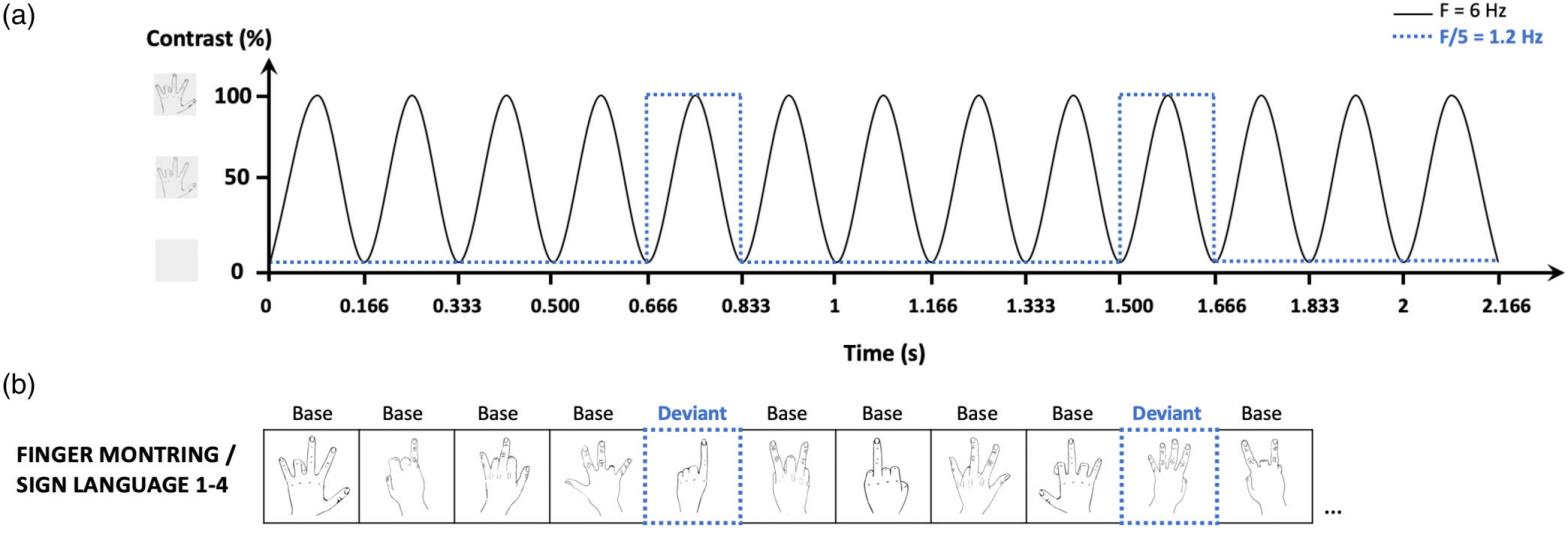
(a) Experimental paradigm of the EEG experiment. Stimulation over time of six stimuli per second (6 Hz) presented with a sinusoidal contrast modulation and deviant stimuli presented at 6 Hz/5 = 1.2 Hz. (b) Examples of the different number representations used in the first EEG experiment. The non‐canonical configurations of numerals (base stimulus) are presented at base frequency and the canonical configurations (deviant stimulus framed with blue dotted lines) are periodically inserted every fifth item.

The present study included two experiments, both of them presenting: (1) non‐canonical finger‐number configurations at 6 Hz as base stimuli, and (2) canonical finger‐number configurations as deviant stimuli at 1.2 Hz (see Figure 2). Sign language configurations (i.e., *signs* for deaf signers but *finger‐montring gestures* for hearing controls) were the deviant stimuli of Experiment 1, while finger‐counting configurations (i.e., *finger‐counting gestures* for both deaf and hearing participants) were the deviant stimuli of Experiment 2. If the linguistic aspect (i.e., *signs*) of canonical hand configurations elicits a more salient processing, then deaf signers should show stronger discrimination responses or different response topographies than the hearing controls in Experiment 1. No group differences should in contrast emerge in Experiment 2 as the finger configurations used as deviant stimuli correspond to finger‐counting gestures in hearing controls as well as in deaf signers.

## 2 EXPERIMENT 1

### 2.1 Methods

#### 2.1.1 Participants

Two groups of Dutch and French‐speaking adults were recruited: a group of 21 congenitally deaf signer adults (9 males, 10 French, Mage = 39.1 years ± 2.92), and a control group of 21 hearing adults who did not know sign language (9 males, 10 French, Mage = 38.8 years ± 3.15) (see Table 1 for a detailed description of the participants). All participants were recruited in Belgium and had normal or corrected‐to‐normal vision and no neurological problems. Hearing controls were matched to deaf participants for age (*F*(1, 40.0) = .006; *p* = .94, *n* ^2^ = .000); gender (*X* ^2^ (1, 42.0) = .000, *p* = 1.00); handedness (*X* ^2^ (1, 42.0) = 1.11, *p* = .29); educational level (*F*(1, 39.0) = .58; *p* = .45, *n* ^2^ = .015); and mother tongue (French vs. Dutch) (*X* ^2^ (1, 42.0) = .000, *p* = 1.00). All deaf participants indicated to be fluent in sign language (*M*_age of acquisition_ ± SE = 6.48 years ± 2.68) and reported it to be their preferred way of communication (for more details, see Supplementary Table 1). Both oral and written instructions in Dutch and in French were given. Additionally, instruction videos in sign language were presented to deaf participants. Participants provided their written informed consent and the procedures were in line with the Declaration of Helsinki. The study was approved by the “Comité d'Ethique hospitalo‐facultaire Saint‐Luc‐UCLouvain” (2019/19AOU/357).

**TABLE 1:** Characteristics of participants

Subject	Age	Sex	Handedness	Onset	Cause	Formal school years (after primary school)
1	56	F	R	0	Hereditary	13
2	47	M	L	0	Rubella	6
3	26	F	R	3 y	Meningitis	12
4	48	M	R	0	O_2_ insufficiency	6
5	51	M	R	0	Meningitis	15
6	28	F	R	0	Genetic	14
7	50	M	L	0	Genetic	7
8	37	F	R	0	Genetic	12
9	49	F	R	0	Rubella	7
10	23	F	R	0	Unknown	9
11	43	M	R	0	Hereditary	5
12	24	F	R	0	Unknown	11
13	20	M	R	0	Unknown	7
14	53	M	R	0	Hereditary	6
15	53	F	R	0	Hereditary	6
16	35	M	R	0	Unknown	9
17	63	F	R	0	Hereditary	N/A
18	35	M	L	0	Genetic	8
19	37	F	R	0	Nerf atrophy	12
20	22	F	R	0	Unknown	10
21	21	F	R	0	CMV	8
22	55	F	R	/	/	11
23	23	M	R	/	/	12
24	23	M	R	/	/	9
25	23	F	R	/	/	10
26	20	F	R	/	/	8
27	38	F	R	/	/	12
28	50	M	R	/	/	11
29	57	F	R	/	/	6
30	46	M	R	/	/	6
31	66	M	R	/	/	11
32	31	M	R	/	/	16
33	57	M	R	/	/	10
34	50	F	R	/	/	9
35	39	M	R	/	/	8
36	38	F	R	/	/	9
37	25	F	R	/	/	14
38	36	F	L	/	/	11
39	49	M	R	/	/	7
40	47	F	R	/	/	7
41	20	F	R	/	/	9
42	21	F	R	/	/	10

*Note*: R = right‐handed; L = left‐handed; F = female; M = male; y = years; CMV = cytomegalovirus.

#### 2.1.2 Stimuli and procedures

##### Stimuli

Non‐canonical hand configurations (i.e., atypical number gestures) representing numbers 1 to 4 were used as the base category (see second line Figure 1), while canonical hand configurations of numbers from 1 to 4 (i.e., *signs* for deaf signers; typical *finger‐montring gestures* for hearing controls) were used as the deviant stimuli (see first line Figure 1). These two sets of stimuli consisted of 48 drawings of finger‐number configurations (including mirror images). Namely, four numbers (1 to 4) designed in three different drawings in original and in mirror orientation were used for the two types of stimuli (base vs. deviant stimuli) (see Figure 1).

##### Procedure

Each sequence consisted in 60 s of stimulation with an additional 2 s of gradual fade in at the beginning and 2 s of gradual fade out at the end of the sequence. Within each sequence, stimuli were presented at a constant frequency rate of 6 Hz (i.e., six images per second, 167 ms per image) by means of sinusoidal modulation of contrast from 0 to 100% (see Figure 2a). Non‐canonical number configurations were used as the base category presented at 6 Hz (see Figure 2b), and every fifth item (6/5 so at a 1.2‐Hz frequency) was a canonical number configuration (i.e., the deviant category). Different drawings of hand configurations were used to ensure that any resulting finding reflects generalization beyond specific visual features. The stimuli in each base/deviant category were randomly presented at the centre of the screen with no immediate repetition of the same stimulus.

Each sequence was repeated three times. A fixation cross appeared at the centre of the screen 2–5 s before the sequence started and stayed at the same position during the entire sequence. To maintain a constant level of attention throughout the stimulation, participants were instructed to focus on a fixation point and to detect brief task‐irrelevant colour‐changes by pressing the space bar (from blue to red, for 200 ms, 10 random colour changes per sequence).

The experiment was created and executed with a software running on JavaScript (Java SE Version 8, Oracle Corporation, USA). The experiment took place in a quiet, low‐lit room. During testing, the participant was seated in front of a table, on which a monitor with an 800 × 600 pixel resolution was placed at a distance of 1 m to display the task.

##### EEG data acquisition and analysis

EEG was recorded at 2048 Hz using the BioSemi Active II system (BioSemi, The Netherlands). Sixty‐four channels were positioned at the standard 10–20 system locations together with four additional posterior electrodes (PO9, I1, I2, PO10). The Common Mode Sense active electrode and the Driven Right Leg passive electrode were used as reference and ground electrodes, respectively. The magnitude of the offset of all electrodes was held below 50 mV.

EEG analyses were carried out using Letswave 5 (https://www.letswave.org/), which is an open‐source toolbox running on MATLAB (The MathWorks, USA). Data files were first resampled to 512 Hz to reduce analysis time, to then pre‐process the files using a Fast Fourier Transform (FFT) band‐pass filter with cut‐off values of .10–100 Hz, and an FFT multinotch filter to attenuate electrical noise at three harmonics of 50 Hz. The fade‐in and fade‐out periods were excluded from the analysis, and channels that showed a high level of artefacts or noise were interpolated (maximum 10% of the channels). Each sequence was then segmented again from stimulation onset until 60 s, in order to contain the largest amount of integer presentation cycles (50 cycles of 1.2 s at 1.2 Hz). Channels were re‐referenced to the common average of all electrodes and the signal from all repetitions of each condition per participant was averaged.

An FFT was computed to obtain the normalized amplitude spectrum for each channel during frequency domain analysis. Frequency resolution of the resulting spectra was .017 Hz (1/60 s), which allows unambiguous identification of the response expected at the frequencies of interest (i.e., 6 Hz for the base stimulation and 1.2 Hz and harmonics for the deviant category detection). Individual FFT data were averaged across participants to allow group analysis. To determine the significant harmonics for both the base (i.e., elicited by the non‐canonical stimulation at 6 Hz) and the deviant (i.e., elicited by the canonical presentation at 1.2 Hz) responses, Z‐scores were computed at every channel. For each discrete frequency bin (x), Z‐scores were calculated as followed: $Z\left(x\right)=\frac{x-\text{mean}\left(\text{noise}\right)}{\text{standard deviation}\left(\text{noise}\right)}$, for which the noise was defined as the 20 surrounding bins of each target bin, excluding the immediately adjacent bins and the extreme (min and max) bins (Lochy & Schiltz, 2019). The number of significant harmonics was determined as the largest chain of consecutive harmonics showing a Z‐score larger than 1.64 (*p* < .050, one‐tailed, testing signal level > noise level).

The signal‐to‐noise ratio (SNR) for the EEG spectrum was computed by dividing the amplitude at each frequency by the average amplitude of 20 surrounding bins (10 on each side) (Liu‐Shuang et al., 2014). Finally, sums of baseline‐corrected amplitudes at the deviant category frequency (1.2 Hz) and significant harmonics (excluding the base stimulation frequency) were obtained to quantify and visualize general topographies of the target response for each participant and per group.

#### 2.1.3 Statistical analyses

Statistical analyses were carried out using IBM SPSS statistics 26 software for Mac OS Monterey 12.6 (Armonk, NY). Statistical significance was set at *p* < .050 for all computations. Data were checked for normality of distribution and presented as mean ± standard error (M ± SE). Amplitude values were transformed using the square root (sqrt) transformation. Results were analysed using a linear mixed model (LMM) with *Amplitude* as dependent variable, *Group* (deaf, hearing controls) and *Hemisphere* (left, right) as fixed factors, as well as considering the *Group* × *Hemisphere* interaction. *Subject* was indicated as random effect, to control for any variability within the groups. First, base rate amplitudes (in μV) were analysed. These involve the visual responses synchronized with the general stimulation frequency (i.e., 6 Hz), that could vary with attention or certain morphological factors. Second, amplitude values (in μV) of the deviant rate were analysed by taking into account the amplitudes of the base rate as covariate. This approach was opted as we compared groups, and differences in brain responses, not specific to our manipulation, can therefore not be excluded. Many studies have indeed already indicated that deaf individuals develop functional and structural brain reorganisation, especially following cross‐modal plasticity (e.g., Alencar et al., 2019; Bottari et al., 2011; Cardin et al., 2020; Dell Ducas et al., 2021; Scott et al., 2014; Vachon et al., 2013). Given that such differences can be due to physiological (e.g., skull thickness, gyri folding, etc.) or even attentional factors, we wanted to control for their potential involvement in the neural responses to the deviant stimuli. Bonferroni post hoc analysis was applied when appropriate to control for family‐wise error rates, while Benjamini–Hochberg corrections were applied to control for false discovery rates (for the latter, see Supplementary Material).

### 2.2 RESULTS

#### 2.2.1 Base rate responses

**FIGURE 3:**
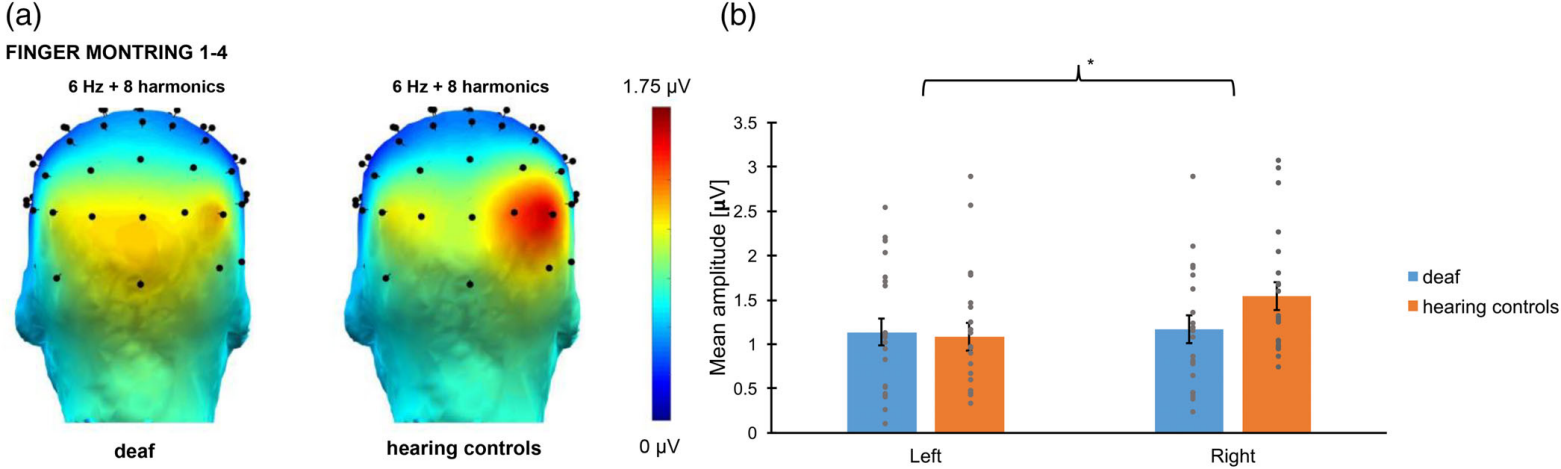
Base responses: (a) Responses elicited at the base rate (i.e., 6 Hz) in Experiment 1 for deaf and hearing controls. Topographies display the sum of amplitude responses for the significant (*Z* > 1.64) harmonics (from 6 Hz to 48 Hz). (b) Bar plot showing the mean amplitudes for deaf (in blue), and hearing controls (in orange) per ROI. Untransformed amplitude values are presented for the sake of clarity. Error bars represent the standard error of the means. Asterisks represent significant difference. Grey points represent individual mean scores.

Responses synchronized with the base frequency (6 Hz) were significant up to eight harmonics (from 6 to 48 Hz). The baseline‐corrected amplitudes were summed and then the responses of the 68 electrodes were ranked. The electrodes showing the highest responses were all located in the parieto‐occipital region (see Figure 3), as observed in our previous FPVS study using similar non‐canonical finger‐number configurations (Marlair et al., 2021). The amplitudes of the channels PO8, O2 and contralateral channels PO7, O1 were averaged into two regions of interest (ROIs). The LMM showed a significant effect of *Hemisphere* (*F*(1, 40.0) = 8.91, *p* = .005) with the right hemisphere showing stronger responses (M ± SE = 1.13 ± .049 μV) than the left hemisphere (M ± SE = 1.00 ± .049 μV). No *Group* effect (*F*(1, 40.0) = .87, *p* = .36) was found, but a significant interaction between *Group* and *Hemisphere* (*F*(1, 40.0) = 4.95, *p* = .032) was observed. Responses in the left hemisphere were not significantly different among groups (M ± SE = 1.01 ± .069 μV for deaf signers, M ± SE = .99 ± .069 μV for hearing controls; *p* = .90), while responses for the right hemisphere were marginally significantly higher for the hearing control group (M ± SE = 1.04 ± .069 μV for deaf signers, M ± SE = 1.22 ± .069 μV for hearing controls; *p* = .074). The *Subject* intercept was significant (*p* = .001). Nevertheless, the inclusion of *Subject* as random effect in the model already controlled for its potential influence on the fixed effects.

#### 2.2.2 Categorical discrimination responses

**FIGURE 4:**
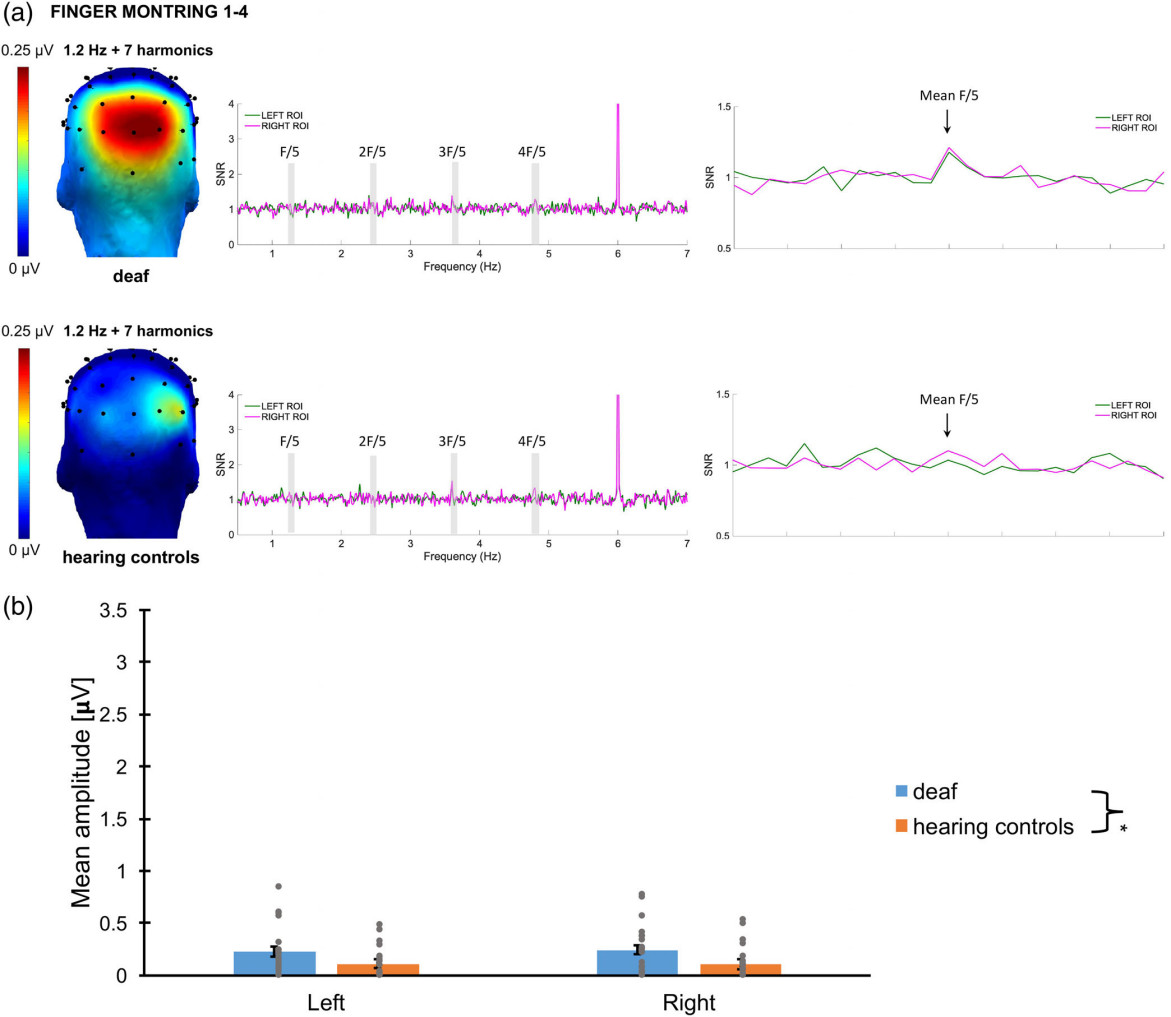
Categorical Responses: (A) responses elicited by canonical detection in the first experiment for deaf and hearing controls. Topographies (on the left) display the sum of amplitude responses for the significant (*Z* > 1.64) harmonics (from 1.2 Hz to 9.6 Hz, excluding the 6‐Hz base frequency). SNR response spectra (middle and right figures) show the electrodes of the ROIs for both conditions: O1, PO3 and O2, PO4. The frequencies of significant harmonics are marked as F/5 (1.2 Hz), 2F/5 (2.4 Hz), 3F/5 (3.6 Hz) and 4F/5 (4.8 Hz). The response at 6 Hz represents the response synchronized with the base stimulation frequency. On the right figure, the averaged SNR of the significant harmonics (excluding the base) is represented centred with 12 surrounding bins on each side. (B) Bar plot showing the mean amplitudes for deaf (in blue), and hearing controls (in orange) per ROI. Untransformed amplitude values are presented by means of clarity, for a base rate reference of 1.00 μV. Error bars represent the standard error of the means. Asterisks represent significant difference. Grey points represent individual mean scores.

Categorical responses elicited by the presentation of canonical configurations at a 1.2 Hz rate were significant from 1.2 to 9.6 Hz (7 harmonics excluding the base stimulation frequency) across the two conditions. Scalp topographies of the sum of significant harmonics suggested posterior bilateral responses (see Figure 4). As shown in several EEG studies examining canonical finger‐number configurations (Soylu et al., 2019; van den Berg et al., 2021, Van den Berg et al., 2022), the channels showing the greatest amplitude of response were located in the parieto‐occipital region. Baseline‐corrected amplitudes were summed and ranked for the 68 channels to identify the channels with the highest responses. Channels O2, PO4 and contralateral channels O1, PO3 were averaged to create right and left ROIs. Statistical analysis indicated no significant *Hemisphere* difference (*F*(1, 43.8) = .13, *p* = .72), nor a significant *Group × Hemisphere* interaction (*F*(1, 42.0) = .079, *p* = .78). There was no significant contribution of the base rate amplitudes (*F*(1, 75.4) = 3.53, *p* = .064), but a significant effect of *Group* (*F*(1, 39.9) = 3.22, *p* = .010). The final model lead to the same conclusion after removing the non‐significant interaction from the analysis: Response amplitudes of deaf participants (M ± SE = .43 ± .044 μV) were significantly higher from those of hearing controls (M ± SE = .26 ± .045 μV, *p* = .010). Note that amplitudes are displayed for a base rate reference at 1.00 μV, and that the S*ubject* intercept was significant (*p* = .007).

### 2.3 Interim discussion

Canonical number configurations from 1 to 4, corresponding to *signs* in deaf individuals and *finger‐montring gestures* in hearing individuals, were periodically inserted in a stream of non‐canonical hand configurations. In this way, the brain responses of deaf signers and hearing controls could be compared. Our results showed a statistically significant effect of *Group*, with deaf participants showing significantly higher responses than hearing controls. This result supports the hypothesis that finger‐montring configurations are processed differently in deaf signers.

## 3 EXPERIMENT 2

### 3.1 Introduction

Results of Experiment 1 could be due to two factors. First, the stronger response amplitudes in deaf individuals could reflect that for them only, the used canonical configurations belong to their linguistic system and have become *signs*. The results could alternatively be merely due to the greater experience deaf signers have with processing hand configurations.

To test these alternative hypotheses, we developed a second EEG experiment using canonical finger‐counting configurations as deviant stimuli. These configurations are canonical, they represent numerosities, but they do not belong to any specific language system. They can thus be considered as *counting gestures* for both groups. If deaf individuals have simply more experience with processing hand configurations, then they should also show stronger responses than hearing individuals using finger‐counting gestures. If results of Experiment 1 were due, however, to the fact that finger‐montring configurations were processed as *signs* of a linguistic system in deaf individuals only, then, no difference should emerge between groups in Experiment 2.

### 3.2 Methods

#### 3.2.1 Participants

The same participants as in Experiment 1 were included in Experiment 2.

#### 3.2.2 Stimuli and procedures

##### Stimuli

**FIGURE 5:**
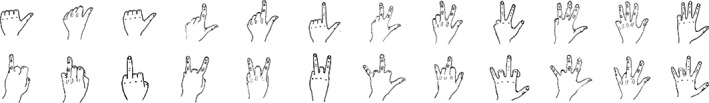
Stimuli that will be used for the different sequences (finger‐counting 1 to 4) of this experiment. First line represents the canonical configurations. Second line represents the non‐canonical ones. Three different drawing designs (and their mirror images) were used for each numerosity.

In the second EEG experiment, non‐canonical hand configurations similar as in Experiment 1 (see second line Figure 5) were used as the base category, while finger‐counting canonical hand configurations from 1 to 4 (typical counting gestures for both groups, see first line Figure 5) were used as the deviant stimuli. The same drawing characteristics and number of drawings were used as in Experiment 1 (see Figure 5).

##### Procedure

The procedure of Experiment 2 was similar to the procedure of Experiment 1 (see Figure 2). Non‐canonical hand configurations (i.e., not typical number gestures for both groups) were again used as the base category presented at 6 Hz (see Figure 2b), but every fifth item (6/5 so at a 1.2‐Hz frequency) was now a canonical finger‐counting configuration (i.e., the deviant category).

##### EEG data acquisition and analysis

Similar procedures for the acquisition and analysis of the data were used for Experiment 2 in comparison to Experiment 1.

### 3.3 RESULTS

#### 3.3.1 Base rate responses

**FIGURE 6:**
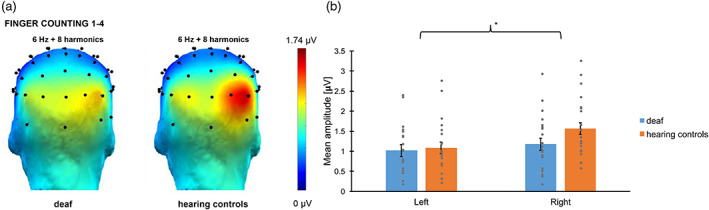
Base responses: (A) Responses elicited at the base rate (i.e., 6 Hz) in Experiment 2 for deaf and hearing controls. Topographies display the sum of amplitude responses for the significant (*Z* > 1.64) harmonics (from 6 Hz to 48 Hz). (B) Bar plot showing the mean amplitudes for deaf (in blue) and hearing controls (in orange) per ROI. Untransformed amplitude values are presented for the sake of clarity. Error bars represent the standard error of the means. Asterisks represent significant difference. Grey points represent individual mean scores.

Responses synchronized with the base frequency (6 Hz) were significant up to eight harmonics (from 6 to 48 Hz). The baseline‐corrected amplitudes were summed and then the responses of the 68 electrodes were ranked. The electrodes showing the highest responses were all located in the parieto‐occipital region (see Figure 6). The amplitudes of the channels PO8, O2 and contralateral channels PO7, O1 were averaged into two ROIs, which are in line with the ROIs found previously for non‐canonical finger configurations in an FPVS study (Marlair et al., 2021). A LMM indicated a significant effect of *Hemisphere* (*F*(1, 40.0) = 11.6, *p* = .002) with the right hemisphere showing stronger responses (M ± SE = 1.13 ± .048 μV) than the left hemisphere (M ± SE = .98 ± .048 μV). No *Group* effect (*F*(1, 40.0) = 1.50, *p* = .23), nor a significant *Group ×* *Hemisphere* interaction (*F*(1, 40.0) = 3.16, *p* = .083) were found. The *Subject* intercept was significant (*p* = .001).

#### 3.3.2 Categorical discrimination responses

**FIGURE 7:**
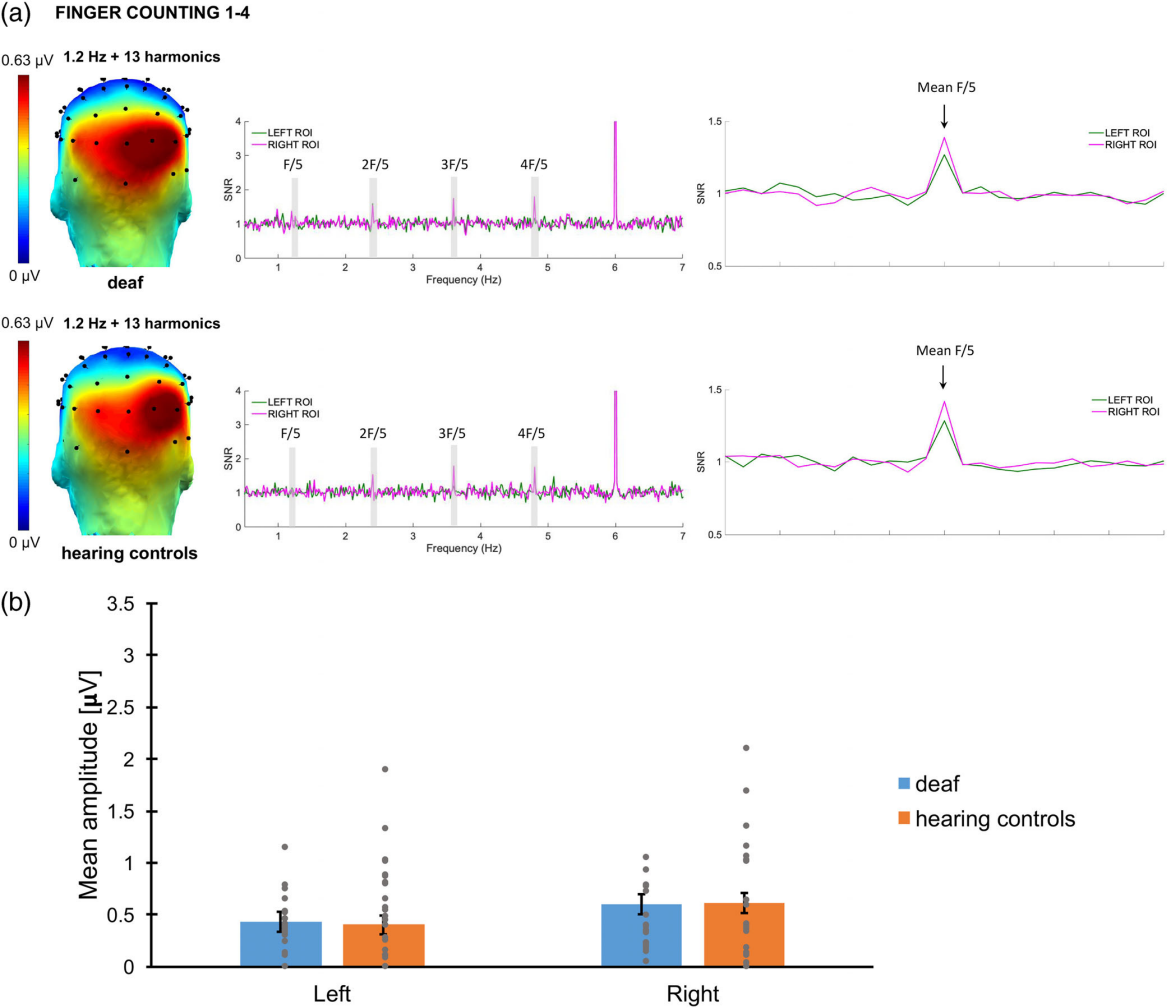
Categorical Responses: (a) responses elicited by canonical detection in the second experiment for deaf and hearing controls. Topographies (on the left) display the sum of amplitude responses for the significant (*Z* > 1.64) harmonics (from 1.2 Hz to 19.2 Hz, excluding the 6‐Hz base frequency). SNR response spectra (middle and right figures) show the electrodes of the ROIs for both conditions: O1, PO7 and O2, PO8. The frequencies of significant harmonics are marked as F/5 (1.2 Hz), 2F/5 (2.4 Hz), 3F/5 (3.6 Hz) and 4F/5 (4.8 Hz). The response at 6 Hz represents the response synchronized with the base stimulation frequency. On the right figure, the averaged SNR of the significant harmonics (excluding the base) is represented centred with 12 surrounding bins on each side. (b) Bar plot showing the mean amplitudes for deaf (in blue) and hearing controls (in orange) per ROI. Untransformed amplitude values are presented by means of clarity, for a base rate reference of 1.00 μV. Error bars represent the standard error of the means. Asterisks represent significant difference. Grey points represent individual mean scores.

Categorical responses elicited by the presentation of canonical configurations at a 1.2 Hz rate were significant from 1.2 to 19.2 Hz (13 harmonics excluding the base stimulation frequency). Scalp topographies of the sum of significant harmonics suggested posterior bilateral responses (see Figure 7). Baseline‐corrected amplitudes were summed and ranked for the 68 channels to identify the channels with the highest responses. In line with other EEG studies including canonical finger‐counting configurations (Marlair et al., 2021; Soylu et al., 2019), the channels showing the greatest amplitude of response were located in the parieto‐occipital region. Channels O2, PO8 and contralateral channels O1, PO7 were averaged to create right and left ROIs. No significant *Hemisphere* difference (*F*(1, 43.4) = .042, *p* = .84), no significant *Group* difference (*F*(1, 39.1) = 1.46, *p* = .23), and no significant *Group × Hemisphere* interaction (*F*(1, 40.2) = .53, *p* = .47) were found. However, a significant contribution of the base rate amplitudes was confirmed (*F*(1, 63.0) = 20.1, *p* < .001). After removing the non‐significant interaction from the model, the final conclusion remained: The right hemisphere (M ± SE = .62 ± .043 μV) showed similar responses as the left hemisphere (M ± SE = .63 ± .044 μV, *p* = .82). No difference between deaf adults (M ± SE = .66 ± .046 μV) and hearing controls (M ± SE = .59 ± .047 μV), *p* = .24 was found. These values are represented for a base rate reference at 1.00 μV. The S*ubject* intercept was not significant (*p* = .37).

### 3.4 Interim discussion

This second control EEG experiment used finger‐counting configurations from 1 to 4. These configurations are not related to sign language, but yet involve numerical information expressed as hand configurations. This might be influenced by the expertise deaf signers have in processing hands. Comparing the neural network underlying finger‐counting configurations however indicated no *Group* difference, which strongly suggests that these configurations are processed in a similar way in all participants. The greater expertise/familiarity that deaf signers present with finger configurations does therefore not play a major role in the discrimination responses observed in the present experiment.

## 4 DISCUSSION

In this study, we wanted to examine whether the brain responses to specific finger‐number representations could be shaped by their belonging to a linguistic system such as sign language. Deaf signer and hearing control adults were requested to perform two EEG FPVS experiments. In Experiment 1, the brain responses underlying finger‐montring number representations (signed numbers) were examined. In this way, the neural network underlying *signs* (for deaf signers) and *finger‐montring gestures* (for hearing controls) was compared. In Experiment 2, the same FPVS paradigm was used to examine the brain responses to finger‐counting configurations (*finger‐counting gestures* for both deaf and hearing participants). In Experiment 1, the typical *finger‐montring gestures* elicited a less salient processing in hearing controls compared to the one observed in deaf signers, which fits with our hypothesis considering that these configurations are processed as *linguistic units* in individuals mastering sign language. As no *Group* difference was highlighted in Experiment 2, we can argue that this effect was not merely due to the experience deaf signers have with processing hand configurations.

In both experiments, the base rate results indicated greater responses in the right hemisphere, which supports a preference of the right hemisphere to numerosities (multiple fMRI studies found specific activation of the right parietal areas during magnitude tasks: Chochon et al., 1999; Eger et al., 2003; Holloway et al., 2010; Kaufmann et al., 2008; Pinel et al., 2001). No *Group* difference in the brain response's amplitudes was highlighted, but a marginally significant *Group* × *Hemisphere* interaction was found in the first experiment. Given that, the base rate response was included as a covariate in our analysis. This allowed us to control for potential differences in attention allocation or morphological factors that could possibly influence the deviant responses, and confirmed that there is a genuine difference between groups on discrimination responses to typical finger‐montring gestures.

The discrimination responses reflect the brain's ability to detect a periodic change that includes both discriminating the deviants from the base stimuli, and generalizing over the deviant's category. The paradigm used therefore measures a differential index of processing (i.e., a response in the context of the base stimuli being used) and not an absolute response to the deviant stimuli. This differential processing was greater in deaf signers than in hearing controls, and we assume that this stems from a stronger neural response to number signs in deaf than in hearing controls. However, we do not know for sure if, as suggested, the deviant stimuli give rise to stronger responses or if the base stimuli give rise to weaker response in deaf signers. Enhanced neural response to canonical in comparison to non‐canonical hand configurations was nevertheless already found in various studies using: (1) canonical finger‐number representations from 1 to 4 (Soylu et al., 2019), (2) canonical number gestures for the numbers 2 to 4 and 7 to 9 during a math verification paradigm (van den Berg et al., 2021), (3) canonical finger‐number configurations showing numerosities 1 to 4 or 6 to 9 during a number comparison task (Van den Berg et al., 2022), and (4) finger‐counting configurations for the numbers 4 to 9 (Marlair et al., 2021). Our results are also in line with previous EEG studies showing brain responses to finger‐number configurations in the parieto‐occipital regions (e.g., Marlair et al., 2021).

Although sign languages (signs anchored in a language system) and non‐linguistic gestures (not anchored in a language system) share the same modalities, only sign languages have established vocabularies and grammatical principles (Grote & Linz, 2003). The distinction between gestures and linguistic signs lies in the fact that gestures are holistic, synthetic and idiosyncratic, while language also contains hierarchical and combinatorics properties (Sandler, 2009). Several studies have already shown that the brain systems engaged in sign language processing differ from those used for non‐linguistic gesture processing. Generating American Sign Language verbs, for example, elicited more activation (using PET data) in the left inferior frontal cortex for deaf signers, while for hearing non‐signers, there was no frontal activation when they generated pantomimes (Emmorey et al., 2011). Pantomime generation, on the other hand, showed more activation in the bilateral superior partial cortex of deaf signers, while hearing non‐signers recruited neural regions associated with episodic memory retrieval (Emmorey et al., 2011). Similarly, Newman et al. (2015) compared how sign languages and non‐linguistic gestures are processed by the brain (using fMRI) in deaf signers and hearing non‐signers. While non‐signers engaged regions involved in human action perception, signers instead engaged left‐lateralized language areas when processing both sign language and gesture. However, sign language activated these language areas more strongly than gestural sequences (Newman et al., 2015). Using a classic visual oddball paradigm (EEG), Deng et al. (2020) examined the neural responses to lexical information of signs in Hong Kong Sign Language in deaf signers versus hearing non‐signers. Deaf signers showed an enhanced visual mismatch negativity brain response to lexical signs. This reflects their activation of long‐term memory traces that facilitate the rapid and implicit retrieval of lexical signs during sign processing. Deaf signers moreover exhibited an enhanced P1‐N170 complex compared to hearing nonsigners across lexical sign and non‐sign standards, which suggests an early neural difference between them (Deng et al., 2020). All of these studies show that sign language representations engage specific brain regions, and that those sign language representations are processed differently by deaf signers compared to hearing non‐signers interpreting gestures.

Although the above presented studies mostly show left‐lateralized responses related to language stimuli, our first experiment triggered the number‐related brain areas in both hemispheres. Previous EEG studies using Arabic digits, which are symbols and have a verbal counterpart in hearing individuals, nevertheless showed right‐lateralized responses (Lochy & Schiltz, 2019; Park et al., 2014; Park et al., 2018). The bilateral responses we observe might stem from the fact that the linguistic aspect (i.e., *sign*) of finger‐montring stimuli was cumulated to its iconic aspect (i.e., *counting gesture*) in deaf signers. Indeed, becoming a symbolic *sign* does not erase the existing analogical link between the number of fingers raised and the numerosity they represent (Berteletti et al., 2021). Nevertheless, we believe that the linguistic aspect of the finger‐montring stimuli is the main factor triggering the highlighted *Group* difference. We therefore argue that the results observed in Experiment 1 reflect a true differential processing because finger‐montring canonical hand configurations of numbers are (linguistic‐iconic) *signs* for deaf signers and only (iconic) *montring gestures* for hearing controls.

In this study, only numbers in the subitizing range were used. A distinction between the subitizing and non‐subitizing ranges is important as fast symbolic—non‐symbolic mapping might only occur for the first four symbols (Carey & Barner, 2019; Le Corre & Carey, 2007; Reynvoet & Sasanguie, 2016; van den Berg et al., 2020). In line with this, a higher right‐parietal P2p response in ERP data to canonical finger‐montring patterns was recently observed for the numbers 1 to 4, but was not elicited by canonical patterns showing numbers larger than 5 (van den Berg et al., 2021; Van den Berg et al., 2022). This could indicate an access to the analogue magnitude representations, but only for the numbers in the subitizing range. Taking into consideration the fact that number signs are part of the language system, it would be interesting to examine whether a difference between deaf and hearing individuals would still be present outside the subitizing range in sign languages that use both hands and the one‐to‐one correspondence principle to sign the numbers 6 to 9 (like the German Sign Language [DGS]). The linguistic aspect of finger‐number configurations might indeed be even more prominent in this case. Applying one‐to‐one correspondence principles may indeed demand more resources in hearing individuals (Marlair et al., 2021), while it might be more automatic (not demanding one‐to‐one mapping) for deaf signers as these are part of their language system (Deng et al., 2020).

Cultural differences between the various existing sign languages could also be used to further test our initial hypothesis. In contrast to the Belgian sign languages, the signed numbers 1 to 10 in DGS partly overlap with the finger‐counting habits of German hearing individuals. If the brain responses to finger‐number representations are indeed shaped by the linguistic versus iconic aspect of these configurations, then opposite results as the ones reported here should be observed in DGS signers. Deaf individuals should, in this case, show more salient responses compared to German hearing controls for the experiment using finger‐counting configurations. In contrast, responses for the finger‐montring experiment might be more similar across groups, considering that finger‐montring configurations are (only) iconic *number gestures* for deaf and hearing German individuals.

Although we linked our findings to the effect of sign language, we cannot exclude the fact that deafness and its following consequences (e.g., brain reorganisation) may also explain part of our data. To disentangle the effects of sign language and auditory deprivation, future studies should examine the brain responses of hearing individuals knowing sign language (late signers or hearing individuals born with deaf parents and having sign language as first language). If sign language knowledge shapes the brain responses to number gestures then similar results should be reported in these groups (with potentially even more salient responses in the group of native signers). A study including hearing signers with varying years of sign language knowledge could moreover shed a light on when exactly hearing signers start to process signed numbers as *signs*.

Finally, as the non‐canonical finger‐number configurations used in our study were more difficult to execute than the canonical ones, one could argue that the neural response observed might only be due to these biomechanical differences (Overmann, 2018). This biomechanical aspect can nevertheless not explain the fact that a *Group* difference was shown in Experiment 1 but not in Experiment 2. Both experiments indeed included the same non‐canonical stimuli. If biomechanical properties of our stimuli were part of the response, then the same neural response would have been observed in both experiments and in both groups.

To sum up, finger‐montring configurations seem to be processed differently by deaf signers as a significant *Group* difference was found in Experiment 1. This difference between deaf signers and hearing controls was not merely due to experience with hand processing, since finger‐counting configurations did not elicit any *Group* differences in Experiment 2. It therefore seems that not only behavioural performances on numerical tasks are influenced by the atypical sensorimotor experiences deaf signers have (Buyle & Crollen, 2022; Buyle et al., 2022), but also the brain correlates underlying finger‐number configurations are shaped by these experiences.

## Supporting information


**SUPPLEMENTARY TABLE 1**. Detailed characteristics of deaf participants

## Data Availability

Correspondence and requests for materials should be addressed to M.B.
