# Supplementary material for: Stronger neural response to canonical finger‐number configurations in deaf compared to hearing adults revealed by FPVS‐EEG
Source: Hum Brain Mapp. 2023 Apr 6;44(9):3555–67. doi: 10.1002/hbm.26297 (PMC10203804; doi:10.1002/hbm.26297)
Supplement: Supplementary file 1 — SUPPLEMENTARY TABLE 1. Detailed characteristics of deaf participants [file HBM-44-3555-s001.pdf]

Supplementary Material: Stronger neural response to canonical finger-number configurations in deaf compared to hearing adults revealed by FPVS-EEG

Supplementary Table 1. *Detailed Characteristics of Deaf Participants*

Subject	Deafness level	Hearing aids	Hearing aids use since	Sign language knowledge	French/Dutch level	Sign language use frequency	Mother tongue	Sign language learned at
**1**	profound	yes	2 y/o	yes	good	daily	French	16 y/o
**2**	profound	yes	6 y/o	yes	good	daily	Dutch	7 y/o
**3**	severe	yes	7 y/o	yes	excellent	daily	French	5 y/o
**4**	profound	no	/	yes	fairly good	daily	VGT	Mother tongue
**5**	profound	CI	2005	yes	excellent	daily	French	20 y/o
**6**	Profound and severe	CI	16 y/o	yes	good	daily	French	19 y/o
**7**	profound	used to have it, not anymore	5 y/o right ear	yes	bad	daily	VGT	Native
**8**	profound	yes	1 y/o but stopped wearing it at 16 y/o	yes	normal	always	LSFB	Native
**9**	profound	Yes, left ear	5 y/o	yes	good	daily	VGT	Mother tongue
**10**	profound	CI, right ear	2,5 y/o	yes	good	daily	VGT	Mother tongue
**11**	profound	Yes, but does not use it often; left ear	/	yes	moderate	always	VGT	Native
**12**	Profound right ear and severe left ear	yes	8 months	yes	good	daily	French	14 y/o
**13**	profound	CI	2 y/o	yes	bon	daily	French	2 y/o
**14**	profound	no	/	yes	does not speak	always	VGT	Native
**15**	profound	no	/	yes	moderate	always	VGT	Native
**16**	profound	yes, left ear	3 y/o	yes	good	daily	VGT	Mother tongue
**17**	profound	no	/	yes	bad	daily	Dutch with gestures	51 y/o
**18**	profound	yes, left ear	since primary school	yes	weak	daily	VGT	Native
**19**	Profound and severe	yes, right ear	11 months	yes	bon	daily	LSFB	Native
**20**	profound	CI	left ear 2,5 y/o; 13 y/o right ear	yes	N/A	daily	French	2 y/o
**21**	profound	CI right, but does	2 y/o	yes	good	daily	LSFB	Mother tongue

	not use it often

Benjamini-Hochberg corrections

To also check false discovery rate errors, Benjamini-Hochberg corrections were moreover performed on the uncorrected p-values obtained in every last statistical model of our analyses. Please note that these corrections result in similar conclusions as reported in the manuscript (using Bonferroni corrections).

Base Rate Responses Finger-montring

Rank (k)	Sorted p-values	P(k) x (m/k)	BH adjusted p-values	Reject H0?
1: intercept	.001	.001 x (4/1) = .004	.004	Yes
2: hemisphere	.005	.005 x (4/2) = .010	.010	Yes
3: group x hemi	.032	.032 x (4/3) = .043	.043	Yes
4: Group	.356	.356 x (4/4) = .356	.356	No

Categorical Discrimination Responses Finger-montring

Rank (k)	Sorted p-values	P(k) x (m/k)	BH adjusted p-values	Reject H0?
1: intercept	.007	.007 x (4/1) = .028	.028	Yes
2: group	.010	.010 x (4/2) = .020	.020	Yes
3: covariate	.050	.050 x (4/3) = .067	.067	No
4: hemisphere	.726	.726 x (4/4) = .726	.726	No

Base Rate Responses Finger-counting

Rank (k)	Sorted p-values	P(k) x (m/k)	BH adjusted p-values	Reject H0?
1: intercept	.001	.001 x (4/1) = .004	.004	Yes
2: hemisphere	.002	.002 x (4/2) = .004	.004	Yes
3: group x hemisphere	.083	.083 x (4/3) = .111	.111	No
4: group	.227	.227 x (4/4) = .227	.227	No

Categorical Discrimination Responses Finger-counting

Rank (k)	Sorted p-values	P(k) x (m/k)	BH adjusted p-values	Reject H0?
1: covariate	.000	.000 x (4/1) = .000	.000	Yes
2: group	.239	.239 x (4/2) = .478	.478	No
3: intercept	.366	.366 x (4/3) = .488	.488	No
4: hemisphere	.817	.817 x (4/4) = .817	.817	No
